# Study on the strength influence mechanism and cracking mechanism of stone powder-cement floor grouting materials

**DOI:** 10.1038/s41598-024-73019-1

**Published:** 2024-10-28

**Authors:** Xinming Chen, Diantao Zheng, Huazhe Jiao, Liuhua Yang, Yangyang Rong, Jinyu Sun

**Affiliations:** 1 Henan Provincial Key Laboratory of Underground Engineering and Disaster Prevention and Control, Henan Polytechnic University, Jiaozuo, 454150 Henan China; 2 School of Civil Engineering, Henan Polytechnic University, Jiaozuo, 454150 Henan China

**Keywords:** Pressure filtration test, Grouting materials, Strength influencing mechanism, Fracture evolution mechanism, Civil engineering, Materials science

## Abstract

Underground grouting is a concealed project, and it is difficult to test the strength of grouting stones in engineering practice. Aiming at the problems of high confined water pressure and strong water-richness of the roadway floor, a pressure filtration test device was developed, and a PFC2D uniaxial compression model was established to study the variation law of stone strength and crack evolution mechanism of grouting materials under different grouting pressures. The results show that with the increase of pressure filtration value, the amount of slurry dehydration increases, the pore water pressure dissipates, the pores between particles are tightly compressed, the contact force between particle skeletons increases, and the strength of stone body increases. Under the condition of the same cement powder ratio (mass ratio of cement to stone powder) or stone powder particle size, the strength of stone body of stone powder-cement grouting material increases with the increase of pressure filtration value. The stress-strain curves of the stone body with different pressure filtration values all experienced three stages: continuous elasticity, fracture expansion and strength failure. Before the peak, the number of cracks increases slowly; after reaching the peak, the micro-cracks extend rapidly and the number increases rapidly, resulting in the final tensile failure of the specimen. This study can provide a basis for the selection of grouting engineering material ratio and grouting pressure parameters.

## Introduction

In the field of civil engineering, grouting is an important method for waterproof plugging and rock and soil reinforcement^1–3^, among which grouting pressure and slurry particle size is an important parameters of grouting. The slurry diffuses deep into the underground aquifer under different grouting pressures, and when the slurry suspension particles are greater than the rock fracture opening, the filtration and dehydration effect will occur^4^. At the fracture end, the slurry suspension particles are tightly stacked with each other, which has a good effect of water blocking and leakage. But the diffusion effect of slurry injected into rock and soil and the strength of stone after grouting are unknown. In order to solve this problem, this paper uses a self-developed stress test device, which can simulate the strength variation of slurry stones under different stress environments.

The pressure filtration dehydration effect runs through the whole grouting process. When the slurry diffuses to the crack mouth with small opening, dehydration occurs, which has a great influence on the strength of the slurry stone body. Chen Yibin^5,6^ conducted indoor grouting tests to compare the strength of cement slurry stone body under normal pressure and high pressure, and found that the strength of cement slurry stone body was significantly higher under high pressure than under normal pressure. Zhang Jingui and Liang Jingwei^7,8^ discovered that the filtration effect exerts a significant influence on the strength of clay cement grout stone bodies. Even when subjected to high water-cement ratios, provided that the filtration effect is robust and prolonged, there will be a substantial enhancement in the strength of grouting stone bodies. Chen Shunman^9^ investigated the influence of curing stress on paste strength and observed a positive correlation between increasing curing pressure and increased strength. In addition to the impact of high pressure conditions on stone body strength, other scholars have explored the influence of coupling factors such as water-cement ratio and grouting rate under high pressure conditions on stone body strength. Fang Kai and Chen Jinxiang^10,11^ investigated the impact of pressure filtration effect on grout spilability and diffusion range. By conducting numerical simulations and comparing with field data, they found that higher grouting pressure and lower water-cement ratio resulted in more pro-nounced filtration press effect of grouting. Zhu Guangxuan^12^ conducted three-dimensional grouting tests to investigate the impact of slurry water-cement ratio and grouting rate on filtration efficiency. The results showed that a lower water-cement ratio led to faster porosity attenuation and more significant filtration effect. Bouchelaghem F^13^ proposed a model for cement slurry penetration under the filter press effect. As high stress gradually weakens the cementing force, interparticle friction affects the cementing body. Additionally, certain researchers have conducted a thorough examination and analysis of the pertinent compaction grouting formulas in order to furnish a relevant foundation for directing practical grouting procedures. Han Weiwei^14^ established a functional relationship between grouting quantity, pore diameter, grouting pressure and slurry water-cement ratio during constant pressure grouting by analyzing test data. The application of grouting materials in water gushing projects has shown good effects on water plugging and leakage prevention. Zou Jian^15,16^ studied the influence of pressure filtration effect on compaction grouting and derived the governing equation for column hole expansion during compaction grouting of saturated clay. Under the same grouting pressure, the greater impact of pressure filtration effect on compaction grouting is observed when effective stress ratio is lower, when slurry thickness is thinner. Wen Shiyou^17^ analyzed the influence range of the average plastic volume strain of saturated clay on the compaction grouting and derived the corresponding calculation formula.

To summarize, the majority of scholars focus on studying the pressure filtration effect of materials primarily based on cement and clay, with little research being conducted on grouting materials that are predominantly composed of stone powder. Although stone powder is a kind of solid waste, it has many excellent properties in mechanical properties, microstructure and hydration mechanism. Compared with the traditional clay grouting material, the stone powder-cement grouting material has the advantages of fast hydration reaction, high early strength, good particle gradation and good mechanical properties of the stone body, which can cope with the complex high ground stress and high grouting pressure environment. As a raw material, stone powder effectively solves the problems of storage, stacking and treatment of stone powder waste, which is not only conducive to environmental and ecological protection, but also conducive to enhancing the commercial value of stone enterprises.

In order to explore the strength change law of slurry stone body of stone powder-cement grouting material under different stress environments, this paper studies the strength change law and crack evolution mechanism of slurry stone body under different stress environments by developing a pressure filtration test device and establishing a PFC2D uniaxial compression model, which provides a basis for the selection of grouting engineering material ratio and grouting pressure parameters.

## Materials and methods

### Materials

**Fig. 1:**
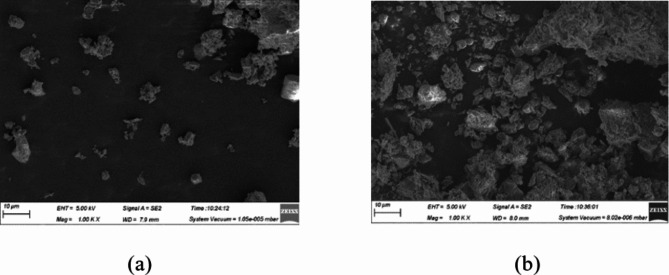
SEM image of cement and stone powder: (**a**) SEM image of cement particles, (**b**) SEM image of stone powder particle.

Cement: P.O 42.5 grade cement produced by Qianye Cement Co., Ltd., Jiaozuo City, Henan Province, China. The constituents of cement are delineated in Table 1. The scanning electron microscope image of cement particles is depicted in Fig. 1a.

Stone powder: Stone powder produced by Jiaozuo Qianye New Material Co., Ltd. The stone powder is grayish white and powdery, and the main chemical composition of the stone powder is shown in Table 2. The scanning electron microscope (SEM) images of stone powder particles are presented in Fig. 1b. The fineness distribution of stone powder was determined using the national standard inspection screen, and the corresponding results are presented in Table 3.

Water: Ordinary tap water.

**Table 1:** Main chemical composition of cement.

Chemical composition	SiO_2_	Al_2_O_3_	CaO	Fe_2_O_3_	MgO	Other
Percentage(%)	20.61	3.98	65.70	2.62	1.56	5.53

**Table 2:** Main chemical components of stone powder.

Chemical composition	CaO	SiO_2_	Al_2_O_3_	MgO	Other
Percentage (%)	43.00	15.72	3.14	1.18	36.96

**Table 3:** Fineness distribution of stone powder.

Stone powder particle size/µm	< 106	106 − 75	75 − 63	63 − 48	48 − 38	38 − 31	> 31
Percentage (%)	6.9	14	18.9	21.8	18	12.6	7.8

### Slurry ratio design

The study investigated the variation in stone strength of stone-cement grouting material under different ratios of Cement-to-stone powder and particle sizes of stone powder. For each grout group, pressure filtration values were tested in five groups (2 MPa, 4 MPa, 6 MPa, 8 MPa, and 10 MPa), with the corresponding mix ratios of grouting materials presented in Table 4.

**Table 4:** Mix proportion of stone powder cement grouting materials.

Test No	Fluidity/cm	Ash powder ratio	Stone powder/g			Cement/g	Water/g
			75 − 63/µm	63 − 48/µm	> 48/µm		
1	30	0.2	499.7	–	–	100.0	541.2
2	30	0.25	400.3			100.0	443.7
3	30	0.3	333.4			100.0	375.0
4	30	0.2	–	499.7	–	100.0	541.2
5	30	0.25		400.3		100.0	443.7
6	30	0.3		333.4		100.0	375.0
7	30	0.2	–	–	499.7	100.0	541.2
8	30	0.25			400.3	100.0	443.7
9	30	0.3			333.4	100.0	375.0

### Pressure filtration equipment

**Fig. 2:**
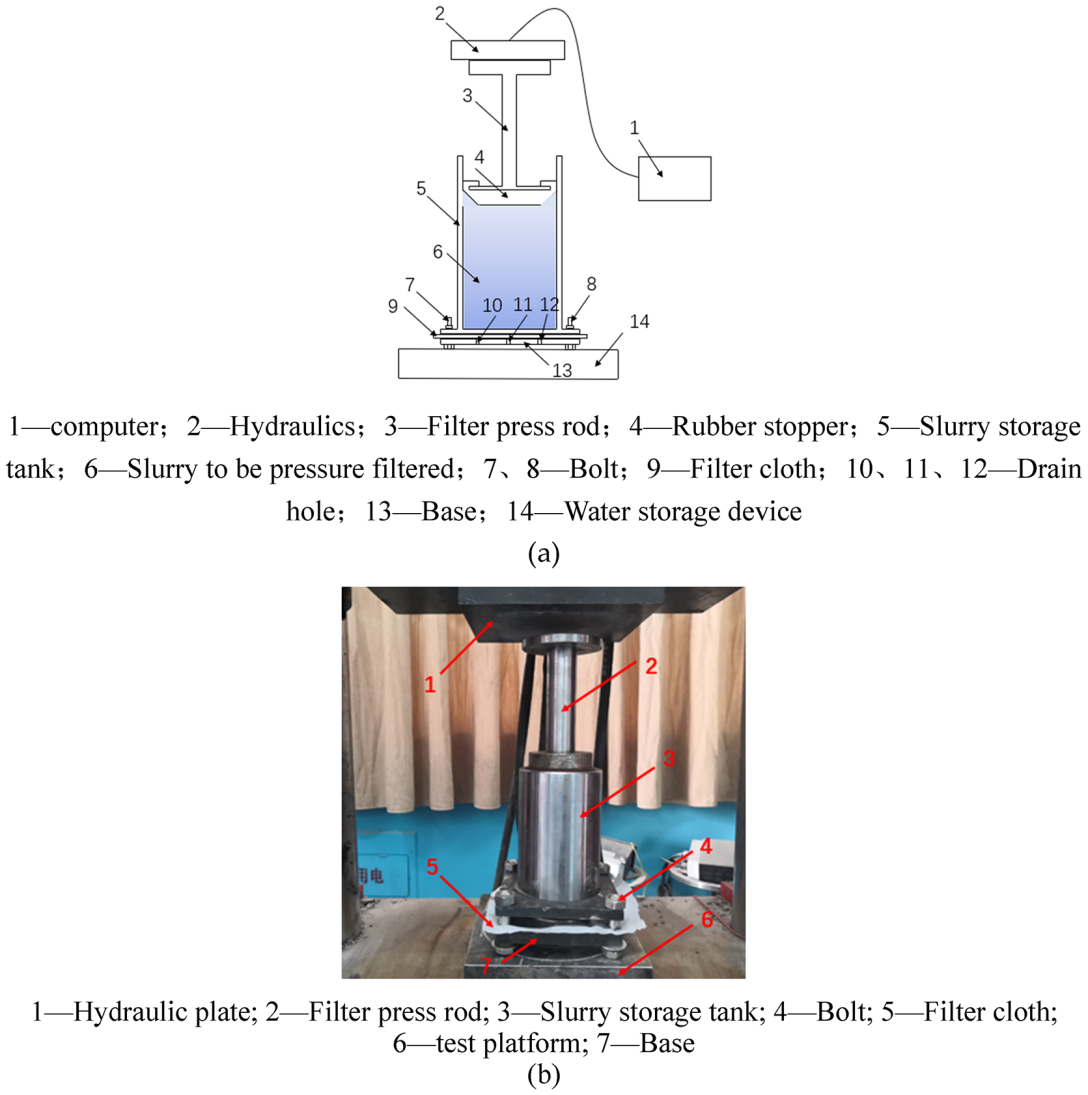
Principle and simulation diagram of pressure filter device: (**a**) Schematic diagram of pressure filter device, (**b**) Model diagram of filter press.

### Uniaxial compressive mechanical properties test method

**Fig. 3:**
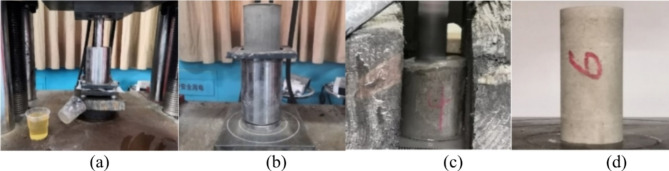
Manufacturing process of cylinder standard specimen: (**a**) pressure filtration process, (**b**) filter cake demoulding, (**c**) borehole sampling, (**d**) cylinder specimen.

The stone powder-cement slurry is poured into the slurry tank and sealed, and the test platform is placed to start the pressure filtration. After the pressure filtration test is completed, the filter cake is subjected to condensation, filter cake demoulding, maintenance, drilling sampling and other operations to complete the production of standard compressive specimens. The process is shown in Fig. 3.

Five different pressure values were utilized in the pressure filtration test, with each group consisting of three distinct ratios of cement-to-stone powder and three varying particle sizes of stone powder. The specimen size was a standard cylinder measuring 25 × 50 mm, while the loading equipment employed was a microcomputer-controlled electronic universal testing machine that operated at a loading speed of 1 mm/min.

## Results and discussion

### Mechanism analysis of pressure filtration effect on the consolidating strength

#### Microstructure to strength mechanism analysis

**Fig. 4:**
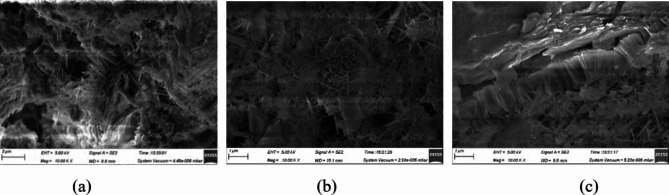
Microstructure of stone body with different pressure filtration values: (**a**) 2 MPa, (**b**) 4 MPa, (**c**) 8 Mpa.

In order to explore the influence mechanism of pressure filtration effect on the strength of stone body, the microstructure of stone body after 10,000 times magnification was observed by electron microscope scanner. In order to ensure significant differences in the microstructure of stone samples subjected to varying filtration pressure values, specimens were selected with filtration pressures of 2 MPa, 4 MPa and 8 MPa after a curing period of 28 days. The resulting microstructures are depicted in Fig. 4.

When the pressure filtration value is 2 MPa, there are many pores inside the stone body, showing an irregular spatial distribution. It is observed that needle-like ettringite grows on the microscopic surface of the stone body, and the structure is relatively loose. When the pressure filtration value is 4 MPa, under the action of pressure, the porosity decreases, the structure is denser, and the flaky hydration products are formed to form a dense network structure, which binds the particles to the cement, and the structure is denser. When the pressure filtration value is 8 MPa, it is observed that the flaky hydration products increase, and the local compact accumulation phenomenon occurs. The cement products are closely bonded to the particles, the pores are further reduced, and the structure is more compact. With the increase of the pressure filtration value, the porosity of the stone body gradually decreases.

#### Analysis of strength mechanism of extrusion drainage

**Fig. 5:**
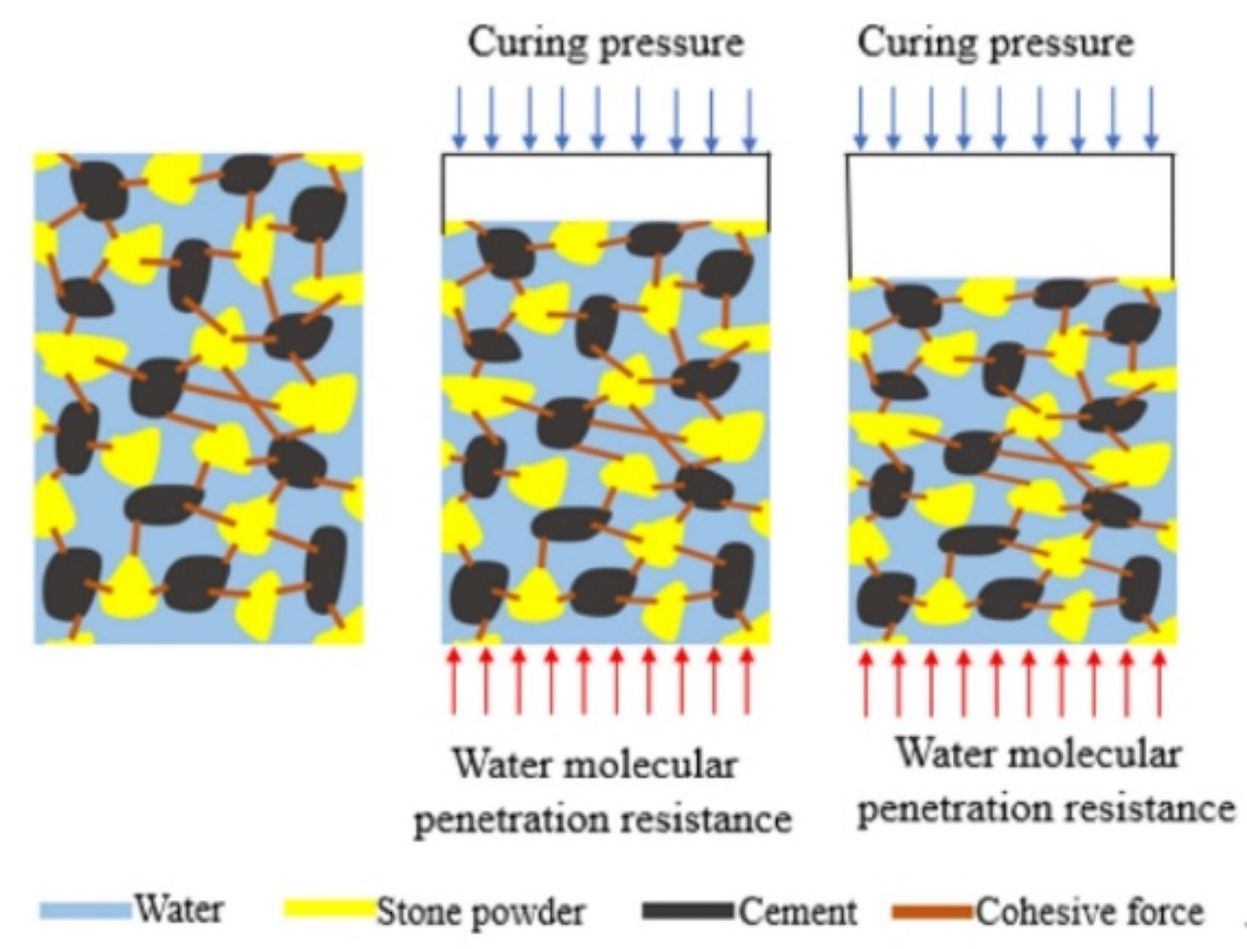
Influence mechanism of filter press dewatering process on strength of stone powder cement.

According to the analysis of the pore structure of the slurry stone body under different pressure filtration values, combined with the extrusion drainage process of the slurry under the pressure filtration effect, the extrusion drainage process diagram of the stone powder-cement slurry under the pressure filtration effect is drawn, as shown in Fig. 5. When the slurry is not filtered, the pores between the particles will be filled with water molecules, forming a curved and connected tubular water channel. In the early stage of the pressure filtration process, under the action of grouting pressure, the thin tube channel is gradually filled with particles, the inter-particle pores are reduced, the compactness is increased, and the initial filter cake is formed. This process is mainly drainage compaction consolidation. As the grouting pressure gradually increases, the slurry is further compacted and consolidated by drainage, the particles are glued together, the skeleton between the particles is continuously compressed, and the thin tube channel is completely closed. When the sum of water penetration resistance and friction force reaches the set value of grouting pressure, the pressure filtration process reaches an equilibrium state. This stage is no longer dehydrated and reaches a stable state. This process is mainly the compaction and consolidation of particle skeleton.

The mechanism of the effect of pressure filtration on the strength of the stone body is mainly as follows: with the increase of the pressure filtration value, the amount of water removed from the slurry increases, the pore water pressure dissipates, the pores between the particles are tightly compressed, the water molecule penetration resistance increases, the contact force between the particle skeletons increases, the slurry force structure improves, and the strength of the stone body increases.

### Analysis of pressure filtration value and compressive strength under different cement ratio conditions

**Fig. 6:**
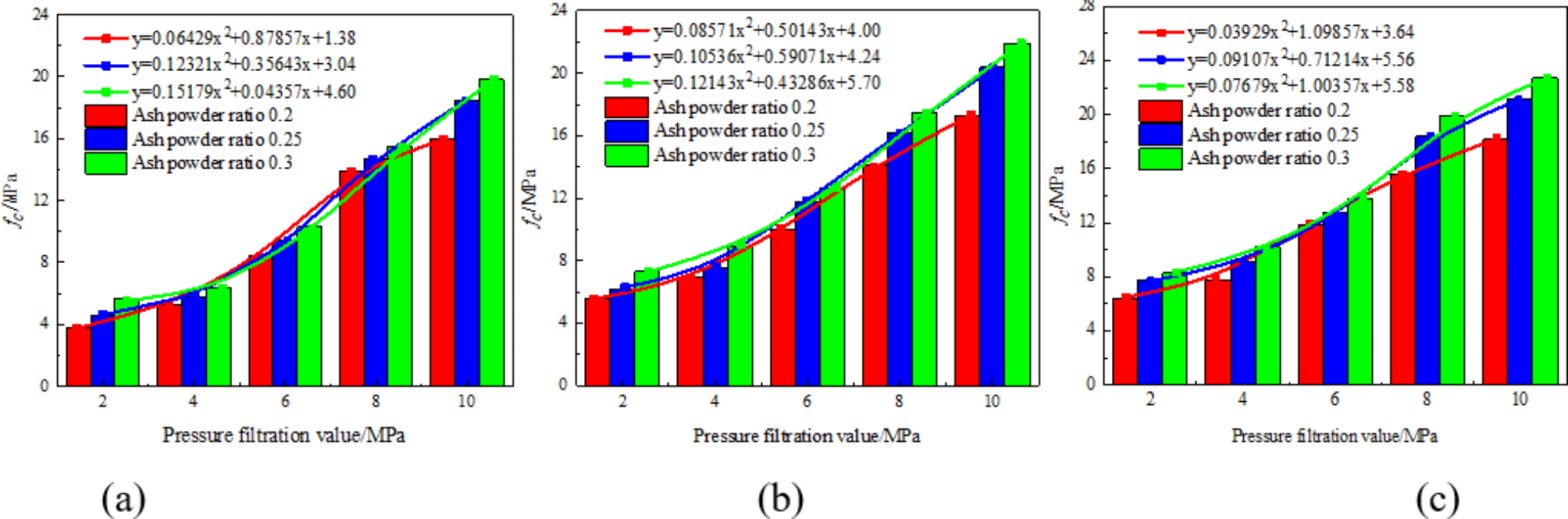
Relationship curve between filter press value and compressive strength under different powder cement ratio: (**a**) The particle size of stone powder is 75 –63 μm, (**b**) The particle size of stone powder is 63 –48 μm, (**c**) The particle size of stone powder is less than 48 μm.

Figure 6 is the change of uniaxial compressive strength of stone body with pressure filtration value of stone powder-cement grouting material with different cement-to-stone powder ratio. Under the condition of the same stone powder particle size, through polynomial fitting, the relationship between the pressure filtration value and the compressive strength of the stone body is a quadratic function of one variable. When the ratio of cement-to-stone powder is 0.3, the fitting coefficient between the pressure filter value and the compressive strength is 0.958; when the cement-to-stone powder ratio is 0.25, the correlation coefficient between filtration value and compressive strength is 0.981. Similarly, when the ash-to-powder ratio is reduced to 0.2, the correlation coefficient increases to 0.989.

After undergoing press filtration, the free water within the grout is expelled, leading to alterations in both the original grout concentration and pore structure, ultimately impacting the uniaxial compressive strength of the grout stone body. The higher the filtration value, the greater the slurry concentration, resulting in reduced porosity and a denser structure, ultimately leading to increased strength of the stone body. According to the microscopic mechanism analysis of the test block, it can be seen that due to the activity of the surface of the stone powder^18–20^, it can react with some cement clinker tricalcium aluminate to form single-carbon hydrated calcium aluminate and semi-carbon hydrated calcium aluminate^21–26^, which prevents ettringite from transforming into monosulfide hydrated calcium sulphoaluminate and enhances the strength of the stone body. The greater the cement-to-powder ratio of the stone body of the stone powder-cement grouting material, the more the amount of cement, the more the number of hydration reaction cements between the cement itself and the cement stone powder, and the higher the strength of the stone body. Therefore, the compressive strength of stone powder-cement slurry with different cement powder ratio is 0.3 > 0.25 > 0.2.

### Analysis of pressure filtration value and compressive strength law under different stone powder particle size conditions

**Fig. 7:**
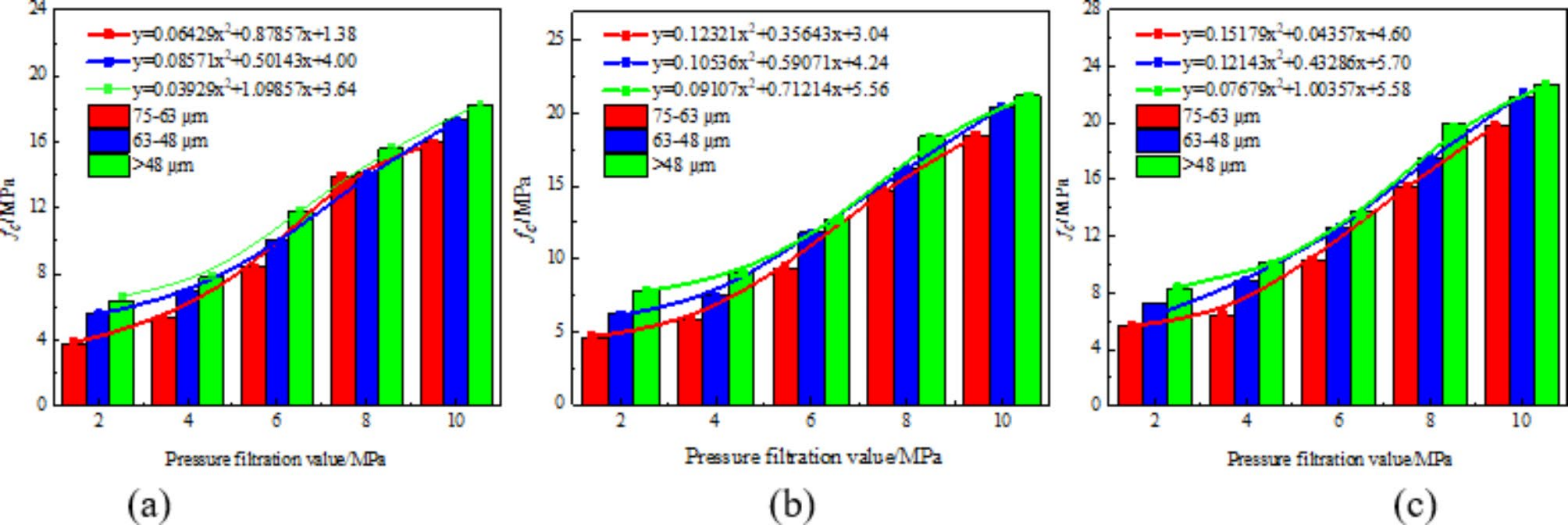
Relationship curve between pressure filtration value and compressive strength under different particle sizes of stone powder: (**a**) the ash powder ratio is 0.20, (**b**) the ash powder ratio is 0.25, (**c**) the ash powder ratio is 0.30.

Figure 7 shows the variation of uniaxial compressive strength of stone powder-cement material stone body with pressure filtration value under different stone powder particle size conditions. Under the condition of the same cement-to-stone powder ratio, through polynomial fitting, the relationship between the pressure filtration value and the compressive strength of the stone body is a quadratic function of one variable. When the particle size of stone powder is 75 –63 μm, the fitting coefficient between the pressure filtration value and the compressive strength is 0.997. When the particle size of stone powder is 63 –48 μm, the fitting coefficient between pressure filtration value and compressive strength is 0.991. When the stone powder particle size is less than 48 μm, the fitting coefficient between the pressure filtration value and the compressive strength is 0.979.

Stone powder can optimize the gradation of slurry particles and has a certain influence on the strength of slurry stones. The main reasons are as follows: stone powder particles are filled in the gap of cement particles, which optimizes the gradation of slurry particles and plays a filling role, so that the porosity of slurry decreases, the compactness increases and the strength increases. The smaller the particle size of the stone powder, the larger the specific surface area, the more water adsorbed, which has a certain water-reducing effect, and the water demand ratio of the stone powder measured by the test is 94.6%, indicating that it has a strong water absorption performance, indirectly reducing the water-cement ratio of the slurry and improving the strength of the slurry stone body. Therefore, when the particle size of the stone powder is less than 48 μm, the compressive strength of the stone body is the largest; when the particle size of stone powder is 75 –63 μm, the compressive strength of stone body is the smallest.

By studying the strength variation of different cement-to-stone powder ratios and different stone powder particle sizes at different pressure filtration values, it can be seen that when the ash-powder ratio is 0.3 and the stone powder particle size is less than 48 μm, the stone powder-cement slurry has the highest strength. The fracture evolution mechanism of the optimally proportioned serous stone body was investigated based on this premise.

### The relationship between stone strength and fracture evolution mechanism was investigated based on PFC2D simulation

#### Build up the compression model

In order to analyze the evolution law of the number of cracks during the failure process of the specimen, a 25 × 50 mm standard model was established by PFC 2D numerical simulation analysis method. The loading mechanism of the model is to set the four-sided wall as the boundary to form a rectangular area. In this area, the model is generated according to the particle radius of cement and stone powder and the slurry ratio, and the normal and tangential bonding strength is applied to the particles, so that the model has the characteristics of compressive strength. Then the walls on both sides are deleted, and the test block model is loaded through the upper and lower walls to realize the uniaxial compression loading simulation test.

**Fig. 8:**
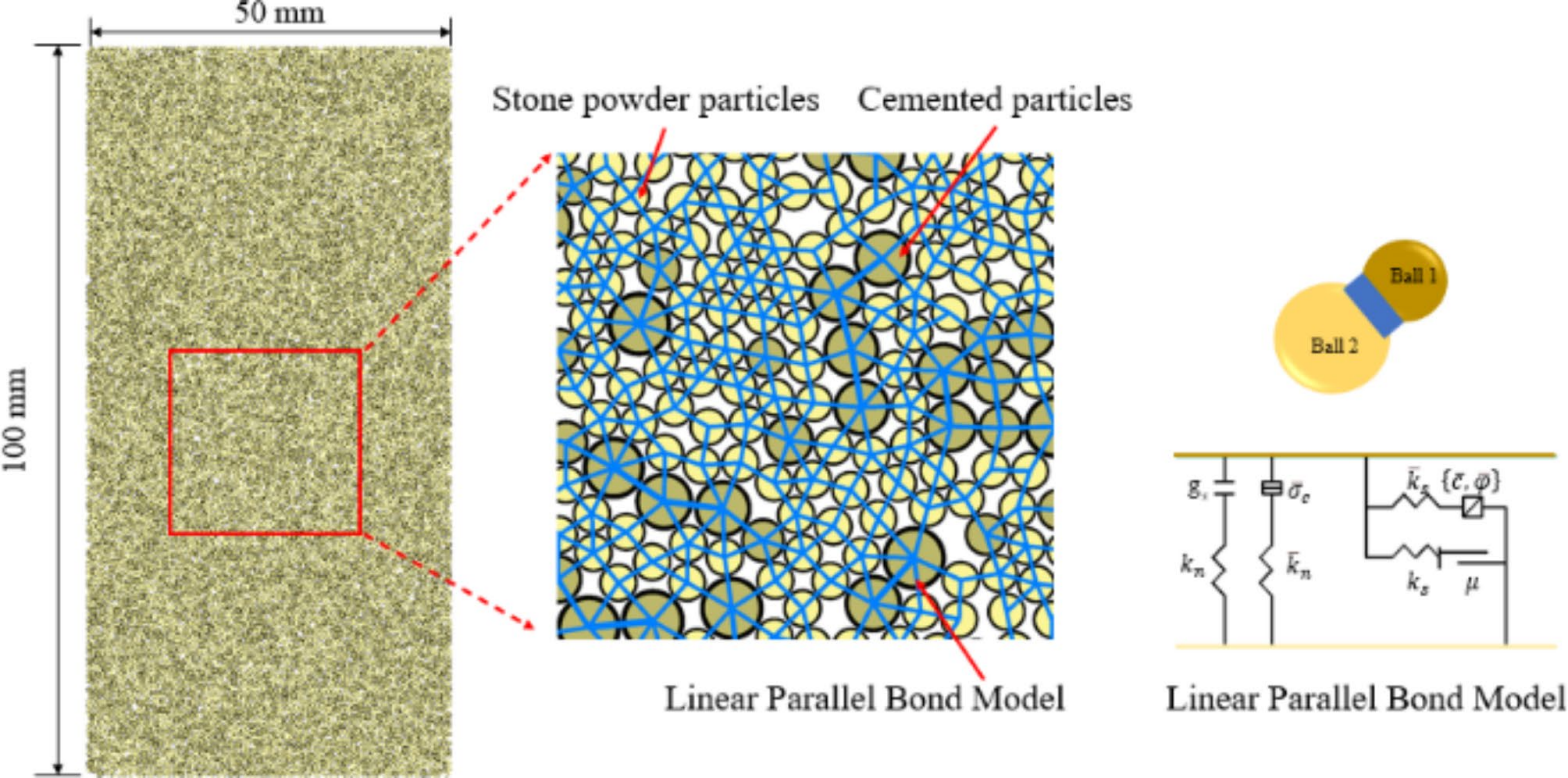
Grouting material particle contact model.

**Fig. 9:**
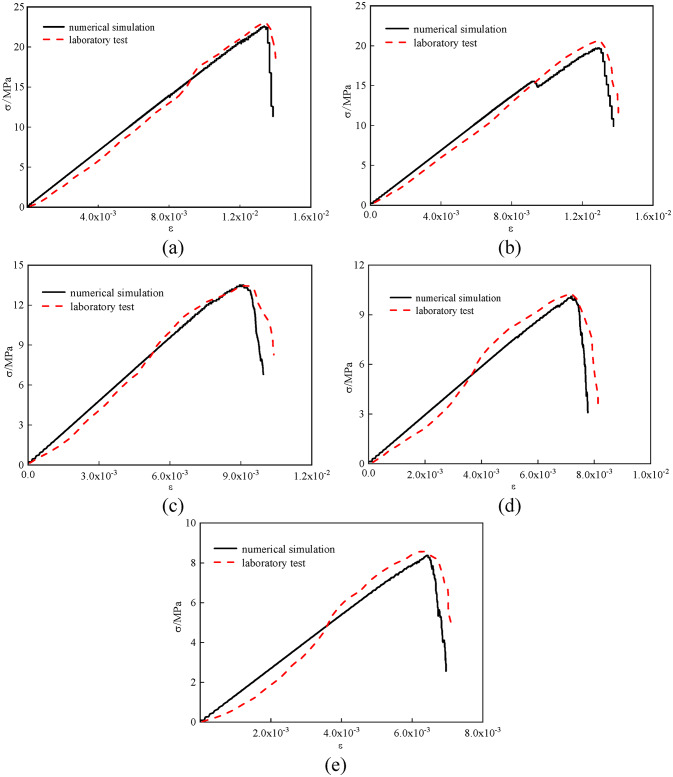
Stress–strain curve and crack number diagram of different pressure filtration values: (**a**) pressure filtration value is 10 MPa, (**b**) pressure filtration value is 8 MPa, (**c**) pressure filtration value is 6 MPa, (**d**) pressure filtration value is 4 MPa, (**e**) Pressure filtration value is 2 Mpa.

The particle meso-parameters are adjusted to generate five groups of models with pressure filtration values of 2 MPa, 4 MPa, 6 MPa, 8 MPa and 10 MPa. The parallel bonding model is used for the stone powder particles and the cementing material. The parallel bond model can simulate the cementing material attached between two adjacent particles. The contact between the sub-layers of the filling body is smooth joint contact, as shown in Fig. 8. In order to make the compressive test model fully correspond to the indoor test process during the loading process, by comparing the stress-strain curves of the numerical simulation process and the indoor test, the values of pb _ emod, pb _ coh and pb _ ten in the meso-mechanical parameters of the model are adjusted to match the stress-strain curves of the indoor test and the simulation test, so as to complete the meso-parameter calibration of the compressive test model. The calibration process is shown in Fig. 9. The micromechanical parameters of each model are shown in Table 5.

**Table 5:** Micromechanical parameters of uniaxial compressive model.

Pressure filtration value	Parallel bond contact				
	pb_emod/(N·m^-1^)	pb_coh/(N·m^-1^)	pb_ten/(N·m^-1^)	fric	UCS/MPa
10 MPa	1.0×10^9^	2.3×10^7^	1.2×10^7^	0.3	21.70
8 MPa	1.0×10^9^	2.0×10^7^	1.0×10^7^		19.70
6 MPa	1.0×10^9^	1.7×10^7^	8.5×10^7^		13.50
4 MPa	1.0×10^9^	1.5×10^7^	7.5×10^7^		10.07
2 MPa	1.0×10^9^	1.4×10^7^	7.1×10^7^		8.38

####  Analysis of stone body strength and crack evolution law

**Fig. 10:**
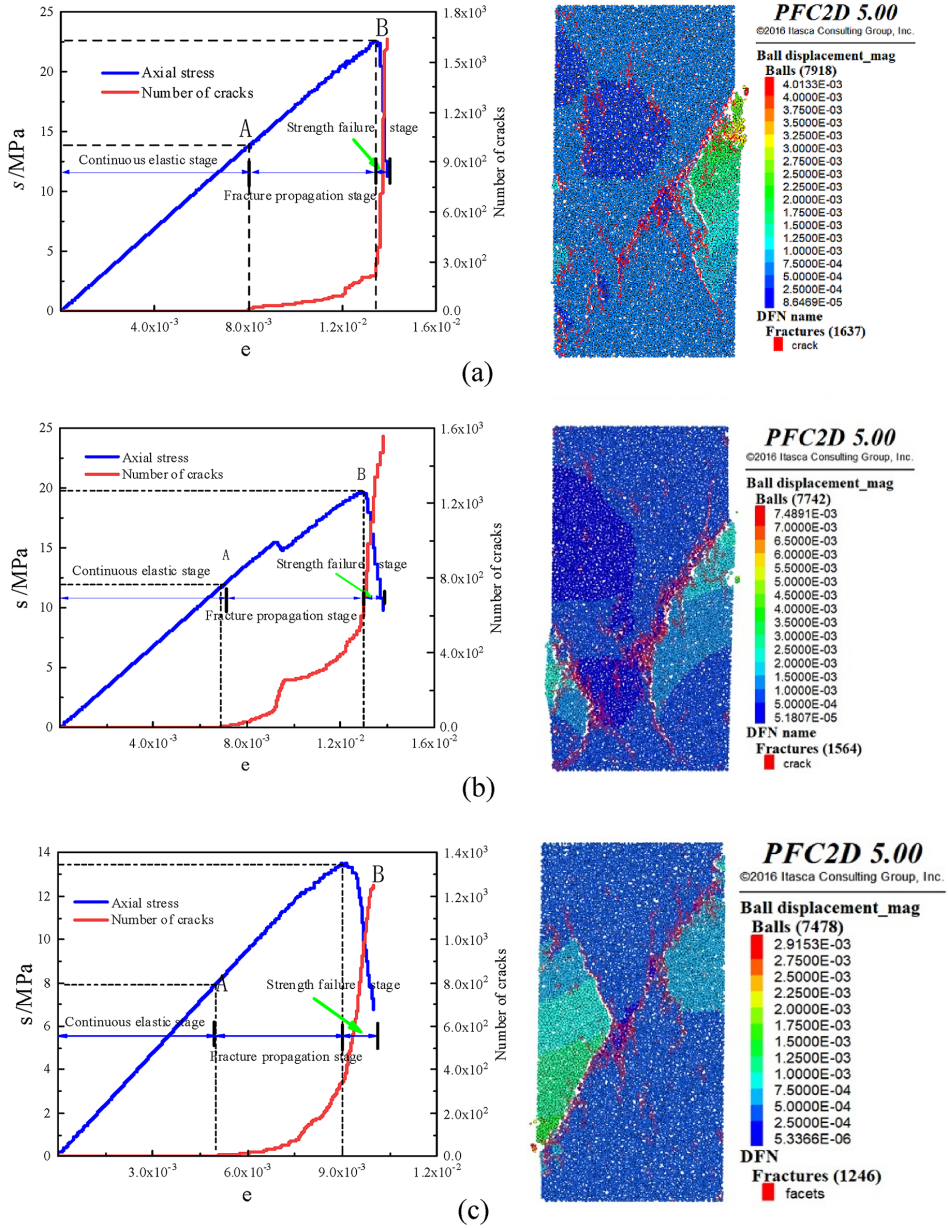

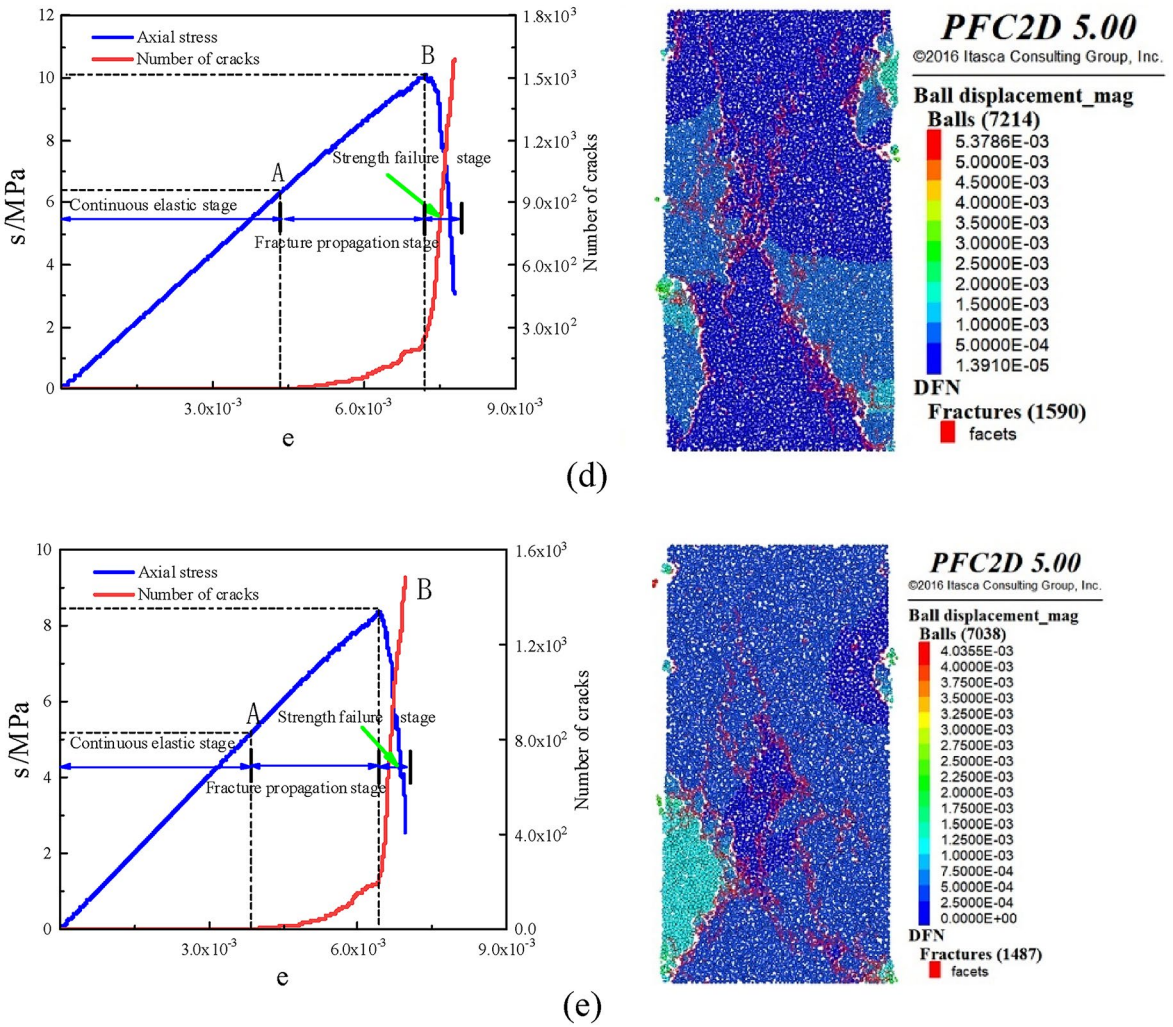
Strength comparison between indoor test and simulation test; (**a**) Pressure filtration value is 10 MPa, (**b**) Pressure filtration value is 8 MPa, (**c**) Pressure filtration value is 6 MPa, (**d**) Pressure filtration value is 4 MPa, (**e**) Pressure filtration value is 2 Mpa.

In order to study the damage evolution mechanism of uniaxial compression fracture of stone powder-cement slurry stone body with different pressure filtration values, a PFC2 D uniaxial compression model was established, and the bond strength between stone powder and cement particles was set. When the contact force between particles is greater than the bond force, cracks will occur between particles. The stress-strain curves and crack evolution curves of slurry stones with different pressure filtration values are shown in Fig. 10.

According to the variation characteristics of stress–strain curve and crack evolution curve of stone body with different pressure filtration values, the failure process of stone body is divided into three continuous processes: continuous elastic stage, crack propagation stage and strength failure stage.

#####  Continuous elastic stage

In this stage, under the action of external load, the internal pores of the stone body are compressed, the distance between particles decreases, and the stress increases with the increase of strain, and the dependent variable is a linear function. At this stage, there is no crack in the stone body, which can be regarded as an ideal elastic material.

##### Stage of crack propagation

Under the continuous action of external loads, cracks within the stone body begin to propagate and stress increases with strain. Cracks initiate when local maximum bond strength is exceeded, primarily concentrated at both ends of the specimen as micro-tensile fractures. The specimen exhibits bearing capacity and remains in a stable development stage.

##### Stage of strength failure

When the in vitro load exceeds the peak stress of the stone body, a sharp drop in stress occurs within the stone body, resulting in loss of bearing capacity and generation of numerous micro-cracks. The main cracks then propagate through the specimen, leading to instability and failure of strength testing.

It can be seen from Fig. 10 that the total number of cracks after the destruction of the stone body is between 1400 and 1600, and the change curve of the number of cracks in the stone body with different pressure filtration values is consistent, which has experienced two stages of slow growth and rapid growth. In the pre-peak stress stage: the number of cracks increases with the increase of compressive strength. At this stage, the stress growth rate is greater than the growth rate of the number of cracks, and the process is accompanied by the formation of micro-tensile cracks. The compressive model is in a stable stage in this process and has the ability to resist external forces ; in the post-peak stage of stress: when the strength peak is reached, the number of cracks increases exponentially and the stress decreases sharply. The cracks increase rapidly and the number increases rapidly, forming a penetrating main crack. In this process, the specimen is in an unstable stage and loses its bearing capacity.

## Conclusion

(1) With the increase of the pressure filtration value, the amount of water removed into the slurry increases, while the pore water pressure dissipates, the pores between the particles are tightly compressed, the water molecule penetration resistance increases, the contact force between the particle skeletons increases, the slurry force structure is improved, and the strength of the stone body increases.

(2) When the cement-to-stone powder ratio or the particle size of stone powder is the same, the strength of stone powder-cement material increases with the increase of pressure filtration value. It is proved by polynomial fitting that the dependent variables conform to the quadratic function relationship. The average fitting coefficient between the ratio of ash to powder and the strength of stone body with different pressure filtration values was 0.976. The average fitting coefficient between the stone powder particle size and the stone body strength with different pressure filtration values was 0.989, and the two variables had a high degree of fitting with the stone body strength with different pressure filtration values.

(3) The failure process of the stone body is divided into three continuous processes : continuous elastic stage, crack propagation stage and strength failure stage. In the early stage of stress peak, the number of cracks increases with the increase of stress, and tensile cracks are generated in this process, and the stone body is in a stable stage. In the later stage of the stress peak, the number of cracks increases exponentially while the stress of the stone body decreases sharply. The cracks extend and the number increases rapidly, forming a penetrating main crack, and the specimen is finally damaged by tension.

## Data Availability

The datasets generated during and/or analyzed during the current study are available from the corresponding author on reasonable request.
